# cGMP in mouse rods: the spatiotemporal dynamics underlying single photon responses

**DOI:** 10.3389/fnmol.2015.00006

**Published:** 2015-03-04

**Authors:** Owen P. Gross, Edward N. Pugh Jr., Marie E. Burns

**Affiliations:** ^1^Center for Neuroscience, University of California DavisDavis, CA, USA; ^2^Departments of Ophthalmology and Vision Science, University of California DavisDavis, CA, USA; ^3^Physiology and Membrane Biology, University of California DavisDavis, CA, USA; ^4^Cell Biology and Human Anatomy, University of California DavisDavis, CA, USA

**Keywords:** phototransduction, photoreceptor, rod, vision, rhodopsin

## Abstract

Vertebrate vision begins when retinal photoreceptors transduce photons into electrical signals that are then relayed to other neurons in the eye, and ultimately to the brain. In rod photoreceptors, transduction of single photons is achieved by a well-understood G-protein cascade that modulates cGMP levels, and in turn, cGMP-sensitive inward current. The spatial extent and depth of the decline in cGMP during the single photon response (SPR) have been major issues in phototransduction research since the discovery that single photons elicit substantial and reproducible changes in membrane current. The spatial profile of cGMP decline during the SPR affects signal gain, and thus may contribute to reduction of trial-to-trial fluctuations in the SPR. Here we summarize the general principles of rod phototransduction, emphasizing recent advances in resolving the spatiotemporal dynamics of cGMP during the SPR.

## OVERVIEW OF ROD PHOTOTRANSDUCTION

The conversion of light energy into electrical signals in rod and cone photoreceptor cells of the retina is the first step in vision. When photoreceptors die, as in diseases such as retinitis pigmentosa or age-related macular degeneration, the otherwise intact visual system loses its normal input, and vision is lost. Fundamental properties of rods and cones, including photon capture efficiency, amplification, kinetics, and spectral sensitivity, strongly constrain the information relayed to the rest of the visual system and ultimately experienced as brightness, form, color, motion, etc.

Night vision under the almost 1000-fold illumination range from starlight to full moonlight operates on a diet literally starved for photons (Burns and Pugh, 2014). All aspects of vision under such nighttime conditions are governed exclusively by signals arising from rods, which generate highly reliable changes in membrane current in response to the absorption of single photons (Baylor et al., 1979b) These single photon responses (SPRs) are driven by a chain of biochemical reactions (“phototransduction”) that transduce photon absorption into changes in the intracellular concentration of cGMP, which through its actions on cGMP-sensitive ion channels enables this exquisite sensitivity to light.

The proteins and signaling pathways underlying rod phototransduction are highly conserved across vertebrates, and in many species the key proteins involved are amenable to efficient biochemical purification and *in vitro* assays. From decades of biochemical work, we know much about the identity, stoichiometries, binding interactions, and even the structure of most of the proteins required for signaling. For example, we know that a photon of appropriate energy excites the G-protein coupled receptor, rhodopsin, which in turn activates many copies of the G-protein transducin (Gα_t_β_1_γ_1_). Each activated Gα_t_ stoichiometrically activates cGMP phosphodiesterase (PDE6), leading to the fall in cGMP concentration. This fall in cGMP causes cyclic nucleotide-gated (CNG) channels on the plasma membrane to close, leading to the reduction of inward cation current (and intracellular free Ca^2+^ levels) and ultimately, membrane hyperpolarization that reduces the synaptic release of glutamate. Timely restoration of the current requires synthesis of cGMP by guanylate cyclases and deactivation of rhodopsin and G-protein/PDE molecules.

The rates of many of these steps can be investigated physiologically in intact rods using suction electrode recording (Baylor et al., 1979a), where the enzymes and substrates are present in their natural concentrations and the membrane current reflects the concentration of cGMP with millisecond precision. With the wide availability of genetically manipulated phototransduction proteins (Fu and Yau, 2007; Burns and Pugh, 2010), mouse rods have become a particularly valuable preparation for investigating the spatiotemporal dynamics of cGMP signaling.

## STRUCTURAL AND BIOCHEMICAL CONSTRAINTS ON cGMP SIGNALING IN RODS

### THE SPATIAL SPREAD OF cGMP SIGNALING IS RESTRICTED BY THE INTRACELLULAR DISKS

#### The nature of the disk stack

**FIGURE 1:**
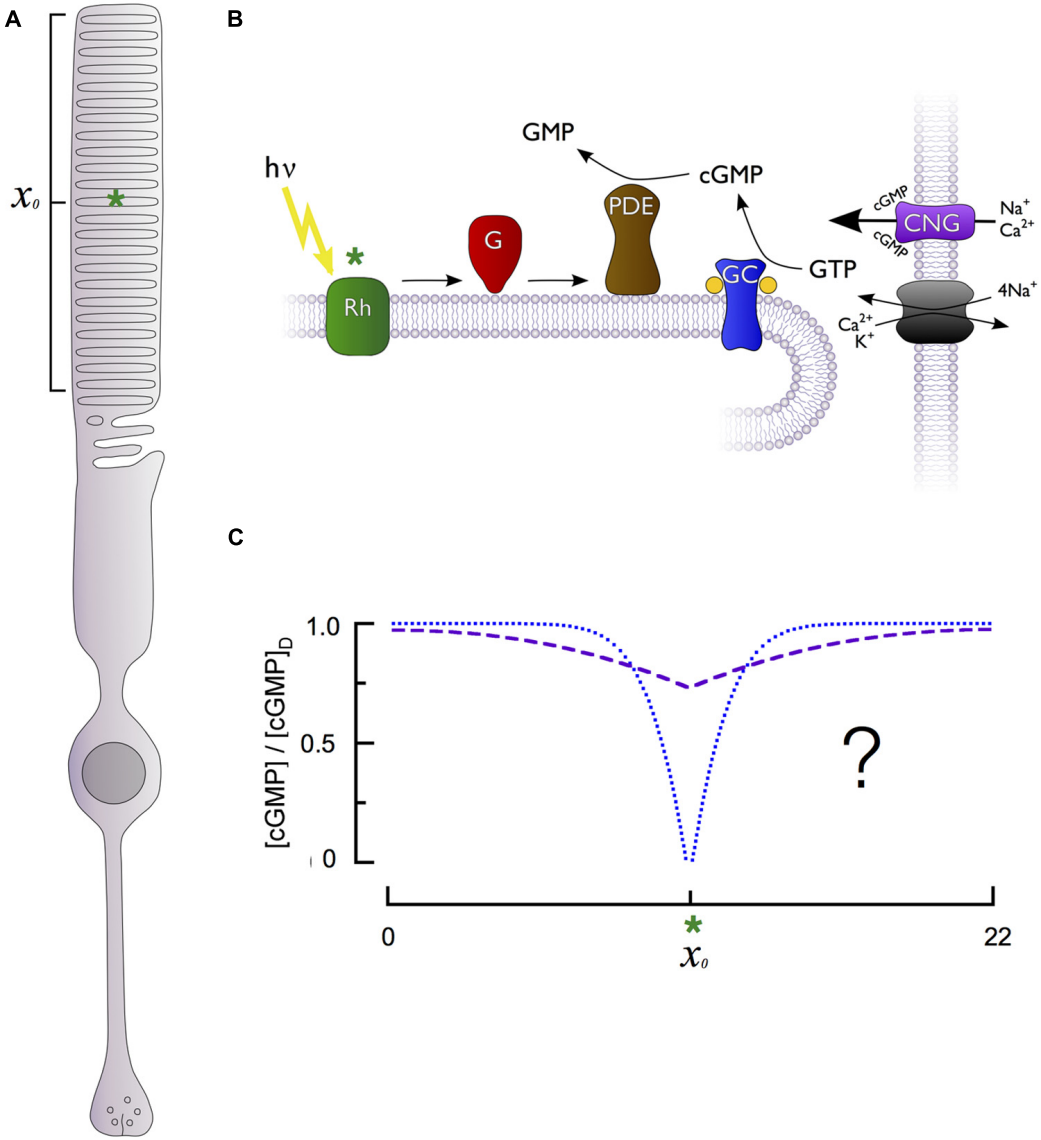
**Generation of cGMP spatiotemporal dynamics by the phototransduction cascade of retinal rods. (A)** Schematic of a rod photoreceptor, highlighting the light-sensitive outer segment compartment (bracket) containing stacks of intracellular membranous disks and a hypothetical photon absorption (green asterisk) occurring in the middle of the outer segment’s length (*x_o_*). **(B)** The primary enzymes responsible for generation of cGMP dynamics are located on the intracellular disk membranes, separated from the cGMP-sensitive channel (CNG) and Na^+^/Ca^2+^, K^+^ exchanger located on the plasma membrane. Photon absorption (green asterisk) by a rhodopsin molecule (Rh) activates multiple G-proteins (G) and cGMP phosphodiesterases (PDE), which are unable to diffuse longitudinally within the rod due to their tight association with the disk membrane. PDE activation in effect produces a point sink for decline in cytoplasmic cGMP along the rod longitudinal axis. The cGMP level is restored by guanylate cyclase activity (GC). **(C)** The fall in cGMP [relative to the dark level, (cGMP)_D_] as a function of distance from the site of photon absorption (*x_o_*, green asterisk) could be either shallow and broad (dashed purple curve) or deep and narrow (dotted blue curve), with markedly different consequences for signal reproducibility and linearity (see text).

Phototransduction occurs within a specialized cylindrical subcellular compartment, the outer segment, which is exclusively devoted to absorbing and transducing photons (**Figure 1A**). The outer segment is filled with a dense stack of protein-rich lipid membranes called disks (**Figure 1B**). The disks house the membrane-associated enzymes of the cascade, including rhodopsin, transducin, phosphodiesterase (PDE), guanylate cyclase as well as regulatory proteins like rhodopsin kinase (GRK1) and the RGS9 complex (below). The abundance of rhodopsin in the disk membranes (25,000–30,000 μm^-2^) and the large number of densely stacked disks (30 μm^-1^) create a high axial absorbance, insuring that a large proportion of incident photons are captured. The density of transducin and PDE is sufficient to insure high diffusional encounter rates, allowing transduction of a single photon to be rapid and strongly amplified (Pugh and Lamb, 1993).

While the primary cascade enzymes – photoexcited rhodopsin (R^∗^), and transducin-activated PDE (E^∗^) – are confined to the disk membrane surface where a photon has been captured, the second messengers cGMP and Ca^2+^ are cytosolic, and can diffuse both radially and axially in the outer segment. Cytosolic diffusion in rods equilibrates much more rapidly in the radial direction than in the axial or longitudinal dimension (Lamb et al., 1981; Olson and Pugh, 1993). As a consequence, diffusion of cGMP in the rod can be characterized by an effective longitudinal diffusion coefficient (*D*_cG_). Because the disks occupy more than 95% of the cross-section of the outer segment, they retard axial diffusion 20-fold or more below its value in unobstructed cytosol (Lamb et al., 1981; Cameron and Pugh, 1990; Olson and Pugh, 1993; Holcman and Korenbrot, 2004). Whether the fall in cGMP is relatively shallow but spatially widespread or deep and spatially restricted (**Figure 1C**) fundamentally affects rod signaling, including the degree to which fluctuations in biochemical processes elicit electrical fluctuations and the range over which rods can linearly sum concurrently absorbed photons. This question has been addressed many times experimentally and theoretically, and the results are summarized below.

Variations in ultrastructure cause *D*_cG_ to vary between the rods of different species. Outer segments of toads and salamanders have far larger diameters (6–15 μm) than those of their mammalian counterparts (1–2 μm). In addition, rod disks have narrow radial gaps called “incisures” that tend to be aligned axially (Cohen, 1963) and greatly facilitate longitudinal diffusion. The length and number of incisures vary across species, ranging from 1 in mouse and human peripheral rods, to 18 or more in tiger salamander rods (Olson and Pugh, 1993).

Estimates of *D*_cG_ have been made from optical measurements of the diffusion of fluorescent compounds, including fluorescein-cGMP (Olson and Pugh, 1993; Holcman and Korenbrot, 2004), and from electrical measurements of cGMP-activated current in dialyzed rods (Cameron and Pugh, 1990; Koutalos et al., 1995a,c; Wu et al., 2006). For the largest diameter rods (salamander), *D*_cG_ was found to be 5–10 μm^2^ s^-1^ (Cameron and Pugh, 1990; Olson and Pugh, 1993), while for narrow mouse rods, ∼40 μm^2^ s^-1^ (Holcman and Korenbrot, 2004; Gross et al., 2012b). *D*_cG_ is, however, only one of several factors governing the spatial profile of cGMP depletion during the SPR, as we now discuss.

### THE SPATIAL EXTENT AND DEPTH OF LOCAL cGMP DEPLETION DURING THE SINGLE PHOTON RESPONSE

Direct measurement of the axial spread of cGMP or Ca^2+^ during the SPR is challenging, because most light used for imaging strongly activates phototransduction. As a consequence, measurements based on the analysis of electrophysiological data have provided the primary body of evidence. Though such measurements are indirect, they are simplified by the lack of voltage-dependence of the rod cGMP channels and the absence of other significant outer segment currents. One experimental approach involves using slits or small spots to deliver light stimuli to different locations in the outer segment while recording the membrane current. Such experiments have yielded conflicting results, with some authors concluding that during the SPR cGMP falls only slightly from its resting level over a large spatial extent (Hemilä and Reuter, 1981; Field and Rieke, 2002), while others concluded that the change is highly localized (Lamb et al., 1981; Gray-Keller et al., 1999). Discrepancies between experiments might be expected because they were performed on rods of different species with distinct outer segment morphology and cGMP diffusion coefficients (see above).

#### A steady-state measure of the spatial spread of cGMP signaling in mouse rods

A recent investigation (Gross et al., 2012b) determined the spatial spread of the cGMP signal initiated by naturally occurring very long-lived “rogue” R^∗^’s, which produce SPRs that are step-like in shape (Baylor et al., 1984). For such SPRs, the spatial profile of cGMP is in steady-state. The cGMP concentration declines as a function of distance from the “point sink” of PDE activity and is predicted by a straightforward analytic expression:

(1) $$\frac{cG\left(x\right)}{cG_{dark}}=\frac{1-e^{-\left|x-x_{0}\right|}/\lambda }{1+C}$$

Here *cG*(*x*) represents the cGMP concentration at axial position *x* along the outer segment_,_ *cG*_dark_ the uniform concentration in the rod in darkness, *x*_0_ the location of photon absorption, λ = $\lambda =\sqrt{D_{cG}/\beta_{dark}}$ the space constant of the cGMP profile, and *C* a constant that depends on known parameters of rod geometry and the measured lifetime of E^∗^. The parameter β_dark_ is the rate constant of spontaneous cGMP hydrolysis in the outer segment in the dark, determined to be 4.1 s^-1^ in mouse rods (Gross et al., 2012b). This mathematical description of the cGMP spatial profile can be converted into the expected change in outer segment current by substituting the well-established relationship between cGMP and channel gating (see Eqs 2 and 3, below) into Eq. 1, and performing spatial integration over the length of the outer segment. Gross et al. (2012b) determined λ and C from experimentally measured, steady-state rogue SPR amplitudes, obtaining λ = 3.1 μm for the space constant and [1/(1+*C*)] = 0.61 for the depth of cGMP decline at *x*_0_. From the same analysis, *D*_cG_ was estimated to be 40 μm^2^ s^-1^, remarkably close to the value 36 μm^2^ s^-1^ estimated for rodent rods by Holcman and Korenbrot (2004) solely from geometric considerations. While this analysis of the spatial profile of cGMP during the SPR was based on the steady-state SPR amplitudes driven by “rogue” rhodopsins, the analysis provides a rigorous lower bound on the depth of the cGMP profile and an upper bound on the spatial extent. It also provides reasonable and consistent estimates of *D*_cG_ and the composite transduction gain (Leskov et al., 2000; Heck and Hofmann, 2001). Most importantly, the resulting values of the parameters β_dark_ and *D*_cG_ are valid generally, and are essential for constraining a spatiotemporal model that incorporates the normal lifetimes of R^∗^ and E^∗^, as well as the effects of calcium feedback regulation to cGMP synthesis, as discussed in the following sections.

### PDE CONTRIBUTES AMPLIFICATION TO THE SPR BUT ALSO SPEEDS SIGNALING AND LIMITS ITS RELIABILITY

R^∗^ is normally active for only a short time (∼40 ms; Gross and Burns, 2010). However, during its brief lifetime R^∗^ activates transducins (G_t_) at a high rate, ∼350 s^-1^ per R^∗^ in mammalian rods at body temperature (Heck and Hofmann, 2001). In turn, each G_t_ activates a PDE, with the result that at the peak of the SPR, ∼10 E^∗^ are active (Gross et al., 2012b). Despite this small number of E^∗^, a sizable change in cGMP concentration is ensured because E^∗^ act upon cGMP in the relatively small volume of the interdiscal space, and each E^∗^ is a highly efficient enzyme. Thus, the PDE catalytic efficiency is *k*_cat_/*K*_m_ = 4.4 × 10^8^ M^-1^ s^-1^ (Leskov et al., 2000), which is close to the rate limit (∼10^9^ M^-1^ s^-1^) set by cGMP diffusion to the PDE catalytic site (Reingruber et al., 2013).

Phosphodiesterase activity affects signaling in other ways, particularly in the dark-adapted rod, because PDE molecules occasionally become spontaneously active. First, this spontaneous or basal “dark” activity sets a threshold that must be overcome by the light-activated PDE activity generated by a single R^∗^. Second, the reciprocal of the basal rate of PDE hydrolysis (1/β_dark_) corresponds to the average lifetime of a cGMP molecule in the dark, and contributes to the speed of SPR recovery (Nikonov et al., 2000; Gross et al., 2012b; Reingruber et al., 2013). Third, the basal hydrolysis rate determines the space constant of the cGMP spatial profile (see above. Eq. 1). Fourth, spontaneous PDE activity produces sizable fluctuations in the membrane current, termed “continuous noise” (Baylor et al., 1980; Rieke and Baylor, 1996), which varies with the membrane density of the PDE in a manner that may compensate for differences in outer segment diameter (Reingruber et al., 2013).

### THE FALL IN cGMP IS SUFFICIENTLY SMALL TO MAXIMIZE THE GAIN CONFERRED BY COOPERATIVE CHANNEL GATING

#### CNG channel properties

Light-stimulated PDE activity decreases the cytoplasmic cGMP concentration, leading to closure of cGMP-gated (CNG) channels in the plasma membrane and reduction of the inward current they carry. The rod CNG channel is a heterotetramer comprising three α- and one β-subunit (Shuart et al., 2011), and is permeable to Na^+^, K^+^, and Ca^2+^ (Craven and Zagotta, 2006). The α-subunit of the channel binds to the Na^+^/Ca^2+^-K^+^ exchanger in the plasma membrane (Schwarzer et al., 2000) via glutamic acid rich protein-like (GARP) domains, likely contributing to the spatiotemporal dynamics of internal calcium. Cytosolic GARP proteins (GARP-1 and GARP-2) are also of fundamental importance in the assembly of the outer segment structure and in stabilizing the disk rims (Korschen et al., 1999; Poetsch et al., 2001; Ritter et al., 2011).

The conductance of CNG channels equilibrates with cGMP concentration within milliseconds (Cobbs and Pugh, 1987; Karpen et al., 1988), so that the time course of the SPR is not limited by the response time of the channel, but rather tracks the changing local cGMP concentration. The cGMP concentration in the dark is 3–4 μM, low relative to the *K_*1/2*_* (∼20 μM) of the channels, so that most CNG channels are closed even in complete darkness. These features of the channel, as well as its relative insensitivity to voltage in the physiological range of membrane potentials (Bodoia and Detwiler, 1985; Baylor and Nunn, 1986), have enabled investigation of phototransduction biochemistry via the electrical response. The gating of the CNG channel by cGMP is cooperative, as captured in the Hill relation that describes the dependence on cGMP of CNG current in a patch of outer segment membrane:

(2) $$\frac{J_{cG}}{J_{\max}}=\frac{cG^{n}}{cG^{n}+{K_{1/2}}^{n}}\,$$

where *J* is current, *cG* is the concentration of cGMP, *n* the Hill coefficient and *K_1/2_* the half-saturating concentration. Early estimates of the Hill coefficient for the channel ranged between 1 and 3 (e.g., Fesenko et al., 1985; Haynes et al., 1986). However, Ruiz et al. (1999) showed that the lower Hill coefficients were likely due to heterogeneity in sensitivity to cGMP (*K_*1/2*_*) across the population of channels in any given patch. This heterogeneity leads to a more shallow dose response curve, resulting in underestimation of the true Hill coefficient. In single channel experiments, the Hill coefficient consistently was measured to be three, a value now widely accepted.

#### Contribution of cooperative gating to gain

In the living rod *cG* << *K_*l/2*_* and so from Eq. 2 the CNG channel current density J_cG_(x)at any point *x* along the outer segment satisfies

(3) $$\frac{J_{cG}\left(x\right)}{J_{dark}}={\left[\frac{cG\left(x\right)}{cG_{dark}}\right]}^{n}$$

where *J*_dark_ is the axial current density in darkness. Thus, the contribution of the cGMP channel’s cooperative gating to the gain of phototransduction is determined by the extent to which the local cGMP level falls during the SPR. If the fractional decline in cGMP is less than about 20%, the change in current will be amplified threefold relative to the fractional change in cGMP concentration. On the other hand, if the local fractional decrease in cGMP exceeds ∼20%, the proportionality between the response amplitude and the overall decline in cGMP will fall short of three, effectively causing “saturation” of the local cGMP-mediated signal. The question of whether or not local saturation contributes to reduction of the trial-to-trial variability of SPRs has been addressed by experiments in toad (Rieke and Baylor, 1998), guinea pig and monkey rods (Field and Rieke, 2002), which concluded that complete local closure of channels is not a major factor limiting variability. In mouse rods, the local fall in cGMP at the peak of the SPR is normally less than 15% of the dark concentration (Gross et al., 2012b) primarily because of the rapid increase in cGMP synthesis, which we now describe.

### CALCIUM FEEDBACK TO cGMP SYNTHESIS

#### Balance between cGMP synthesis and hydrolysis

In darkness there is a balance between cGMP synthesis and hydrolysis, leading to a steady level of cGMP (cG_dark_ in Eq. 3). With the rate of synthesis identified as α_dark_ and the rate constant of hydrolysis as β_dark_ (see above), the steady-state cGMP concentration in darkness must satisfy

(4) $$cG_{dark}=\alpha_{dark}/\beta_{dark}$$

The basal rate of cGMP synthesis has been estimated from biochemical assays to be between 9 and 24 μM s^-1^ in dark-adapted mouse rod outer segments (Makino et al., 2008). Given β_dark_ = 4.1 s^-1^ (Gross et al., 2012b), a synthesis rate of 9 μM s^-1^ corresponds to *cG*_dark_ = 2.4 μM, while that of 24 μM s^-1^ corresponds to 6.6 μM. The former of these values is close to that (3.2 μM) estimated from experiments in salamander rods (Cameron and Pugh, 1990).

#### Activation of guanylate cyclase by declining calcium

Activation of rhodopsin by light adjusts the equilibrium level of cGMP by stimulating not only cGMP hydrolysis, but also synthesis in an intricate feedback involving Ca^2+^. In darkness about 15% of the inward current through the CNG channels is carried by Ca^2+^, which is homeostatically pumped out by the Na/Ca-K exchanger (NCKX): closure of channels rapidly causes internal Ca^2+^ to decline as its influx decreases and extrusion by NCKX exchange continues. Following light stimulation, recovery to the dark-adapted state requires not only deactivation of R^∗^ and E^∗^, but also restoration of the dark concentration of cGMP, which is synthesized from GTP by retinal guanylate cyclase 1 and 2 (RetGC-1 and RetGC-2, “GC”; for review see Sharma, 2010). The rate of cGMP synthesis depends strongly on intracellular calcium concentration (Koch and Stryer, 1988; Koutalos et al., 1995b). This calcium dependence is conferred by guanylate cyclase activating proteins (GCAP-1 and GCAP-2), which are inhibited by calcium binding (Palczewski et al., 1994; Dizhoor and Hurley, 1996, 1999; Ames et al., 1999) but disinhibited as calcium declines during the light response.

The calcium dependence of cyclase activation by GCAPs follows a Hill relation with a Hill coefficient of ∼2 and an effective K_*1/2*_ between 60 and 130 nM (Dizhoor and Hurley, 1996; Ames et al., 1999; Palczewski et al., 2000; Makino et al., 2008; Peshenko et al., 2011). Mechanistically, calcium sensitivity is conferred by three functional EF-hands, while a fourth EF-hand does not bind calcium. In the dark-adapted outer segment when Ca^2+^ is at its highest level, metal binding sites are occupied by calcium, and GCAPs are inhibited from activating GC. As Ca^2+^ falls during the light response, these binding sites become occupied instead by Mg^2+^, which facilitates GC activation (Peshenko and Dizhoor, 2004). The Ca^2+^/Mg^2+^ sensor properties exhibit slightly different sensitivities for GCAP-1 and GCAP-2, likely contributing to their differential effects on the light response (Dizhoor et al., 2010).

#### Cyclase activation in living rods

The time course with which Ca^2+^ declines when channels close depends on the rate and *K*_*1/2*_ of the Na^+^/Ca^2+^-K^+^ exchanger (NCKX), the volume the outer segment, the calcium buffering capacity and the diffusion coefficient (Lagnado et al., 1992). In large amphibian rods the decrease in calcium estimated from the exchange current has a principal time constant of ∼2 s (Cobbs and Pugh, 1987; Hodgkin et al., 1987), while in small mammalian rods the decline is more than 10-fold faster (Makino et al., 2004). Measurements of the concentration of calcium of the outer segment in the dark in rods of different species range from to 250 nM (mouse, Woodruff et al., 2002), to 273 nM (toad, Korenbrot and Miller, 1989), to 670 nM (salamander, Sampath et al., 1998).

**FIGURE 2:**
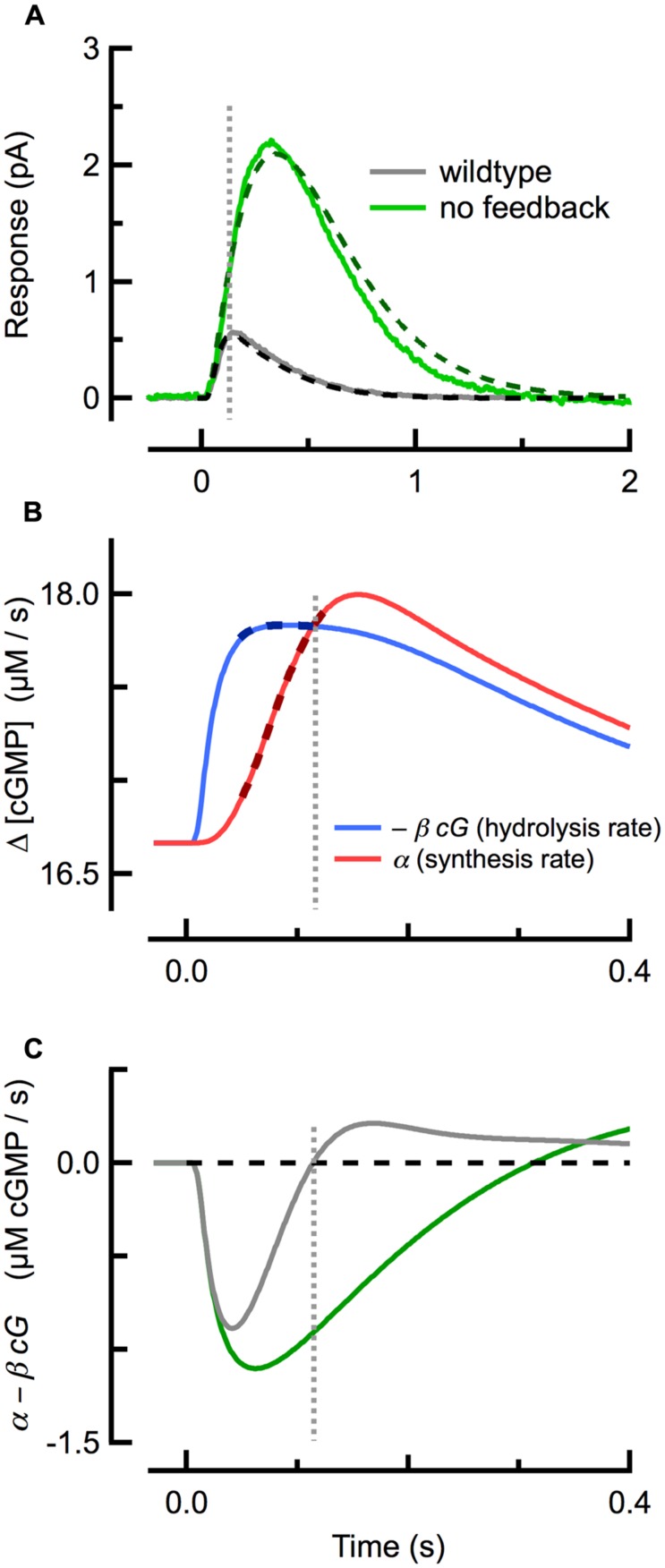
**Delayed, ramping cGMP synthesis contributes to the temporal precision and reproducible amplitude of the SPR. (A)** Measured (solid) and simulated (dashed) SPRs for WT (gray) and GCAPs^-/-^ (green; no calcium feedback) rods originally published in Gross et al. (2012b). **(B)** Spatially integrated rates of cGMP hydrolysis (blue trace) and synthesis (red) during during the WT SPR, as computed with the spatiotemporal model fitting the WT trace in **(A)**. The rate of cGMP hydrolysis plateaus quickly, while the rate of cGMP synthesis exhibits a brief delay followed by a slower, ramping climb to its maximum. Thick dashed lines indicate the coincident plateau and ramping phases of hydrolysis and synthesis, respectively. **(C)** The difference between the cGMP synthesis and hydrolysis rates for WT and GCAPs^-/-^ rods. The rate of cGMP synthesis in GCAPs^-/-^ rods remains constant (not shown) due to the absence of calcium feedback. Note that the zero-crossings of the traces in **(C)** correspond to the SPR peaks in **(A)**. Vertical dashed line indicates time of WT SPR peak in all panels.

Strong cyclase activation occurs even during the SPR, restricting its amplitude and speeding its recovery: in mouse rods lacking calcium feedback activation of cyclase (GCAPs^-/-^) the SPR has an approximately three- to fourfold greater amplitude than that of WT rods, reaching its peak amplitude and recovering much more slowly (**Figure 2A**; Mendez et al., 2001; Burns et al., 2002). While the increased amplitude of SPRs of GCAPs^-/-^ rods results primarily from the absence of calcium feedback to cyclase, the slowed recovery is determined by the time constant of cGMP turnover (1/β_dark_ = 245 ms), which becomes the rate-limiting step of recovery (see above; Nikonov et al., 2000; Gross et al., 2012b).

## INSIGHTS FROM A SPATIOTEMPORAL MODEL OF cGMP AND Ca^2+^ DYNAMICS

### VALUE OF MATHEMATICAL MODELS OF THE SINGLE PHOTON RESPONSE

Mathematical models can contribute much to the understanding of cellular dynamics by compactly embodying current molecular knowledge, by yielding quantitative insight into key biological functions such signal gain and reliability, and by providing explanations of malfunction in disease or molecularly manipulated states (e.g., Boyett et al., 2000; Tao et al., 2011). Models of phototransduction have contributed, for example, to the understanding of the molecular mechanisms of amplification of the light response (Pugh and Lamb, 1993). For many years it has been understood and generally accepted that the entire time course of the rod light response should be describable in terms of a pair of coupled partial differential equations governing cytoplasmic cGMP and Ca^2+^ with initial and boundary conditions, and with an appropriate kinetic model of R^∗^ and E^∗^ activation and inactivation (Lamb and Pugh, 1992; Andreucci et al., 2003). The SPR has been an important target of this modeling, and perhaps no feature of the SPR has been more celebrated and yet more difficult to understand in molecular terms than its stereotypic amplitude: specifically, the measured coefficient of variation of the amplitude (SD/mean) is 0.2–0.4 (Baylor et al., 1979b; Rieke and Baylor, 1998; Whitlock and Lamb, 1999; Field and Rieke, 2002; Hamer et al., 2003; Gross et al., 2012a), much lower than that (1.0) that would occur were the single R^∗^ underlying it to deactivate in a single stochastic step. Several models of the SPR have been published that address this issue (Rieke and Baylor, 1998; Field and Rieke, 2002; Hamer et al., 2003), including several spatiotemporal models, i.e., models that include the diffusion of cGMP and Ca^2+^ (e.g., Andreucci et al., 2003; Bisegna et al., 2008; Reingruber and Holcman, 2008; Caruso et al., 2010, 2011; Gross et al., 2012a; Reingruber et al., 2013). In the following paragraphs we summarize some novel results that have come from developing and applying a “fully constrained” spatiotemporal model of phototransduction to the SPR of mouse rods with specific molecular perturbations to the phototransduction machinery (Gross et al., 2012a,b), including fresh insight into the molecular mechanisms underlying its stereotypic amplitude. First, we state the principles, briefly explaining what is meant by “fully constrained.”

### GROUNDING THE MODEL IN BIOCHEMICAL AND BIOPHYSICAL MEASUREMENTS AND CONSTRAINTS

Given that solutions to the governing equations can be produced that adequately fit rod photoresponses, a critical question to be addressed before valid inferences can be drawn is “Are the parameters of the model well constrained?” Ill-constrained models, even if they accurately describe aspects of the data, can lead to ambiguous and even false inferences. Thus, every potential constraint from structure, biochemistry and experiment independent of the fitting process should be incorporated, such that in the ideal there are no truly “free parameters,” i.e., parameters whose values are determined solely by fitting.

As described above, the key spatial parameter *D*_cG_, rate parameter β_dark_, and the composite transduction gain were determined from independent measurements (Gross et al., 2012b). The *in situ* lifetimes of the transduction amplifiers (R^∗^ and E^∗^) were incorporated from previous electrophysiological measurements of bright flash responses in genetically targeted mice (Krispel et al., 2006; Gross and Burns, 2010), and kinetic parameters relating to Ca^2+^-dependent activation of NCKX and GCAPs were taken directly from biochemical studies. Dark and maximal cyclase activation levels were determined from experiments and analysis independent of fitting the shape of the SPR. The average lifetimes *in situ* of the amplifying enzymes R^∗^ and the PDE active complex (E^∗^) were determined from independent measurements from bright flash responses of genetically targeted mice.

The rods of molecularly targeted mice provide a rich body of individual constraints on model parameters. One of the most important such constraints came from rods lacking GCAPs: the absence of GCAPs completely eliminates calcium feedback to GC and thus uncouples parameters governing calcium dynamics from the rest of the phototransduction equations. Requiring the model of WT SPRs to employ precisely the same parameters as used to describe GCAPs^-/-^ SPRs is highly constraining and informative (Gross et al., 2012b), as the values for a only few parameters involved in calcium fluxes and buffering could then be constrained within biochemically determined limits, and then optimized by the fitting process.

### CALCIUM-FEEDBACK ACTIVATED cGMP SYNTHESIS CONFERS TEMPORAL PRECISION AND AMPLITUDE STABILITY TO THE SPR

Several studies had previously concluded that calcium feedback to cGMP synthesis does not play a role in the reproducibility of the SPR (Rieke and Baylor, 1998; Whitlock and Lamb, 1999), but this conclusion has proven to be inconsistent with work showing that reproducibility is degraded in GCAPs^-/-^ rods (Gross et al., 2012a). Investigation of the underlying cGMP dynamics using a spatiotemporal model revealed that a delay in cyclase activation relative to cGMP hydrolysis plays a critical role. At the beginning of the SPR (**Figure 2A**), the rate of light-driven cGMP hydrolysis increases rapidly (**Figure 2B**; blue), after which it begins to plateau as Ca^2+^ declines steadily and the rate of calcium-sensitive cGMP synthesis rises along a ramp (**Figure 2B**; red). The steepness of the synthesis ramp is roughly proportional to the plateau of the hydrolysis rate, so that the ramp overtakes the plateau of hydrolysis at nearly the same time, almost independent of the R^∗^ lifetime (Gross et al., 2012a). This equilibrium between cGMP hydrolysis and synthesis corresponds to a net rate of change of cGMP of zero (**Figure 2C**) and so to the peak of the SPR. Thus, delayed, ramping calcium feedback activation of cyclase confers invariance to the SPR time-to-peak in mice with altered R^∗^ and E^∗^ lifetimes, and a result, to their SPR amplitudes.

### CALCIUM FEEDBACK CONTRIBUTES SUBSTANTIALLY TO THE REPRODUCIBILITY OF THE SPR

It has long been hypothesized that late steps in biochemically feasible R^∗^ deactivation would greatly decrease SPR reliability “at late times,” and that the only possible explanation for the observed high reliability would be a large number of small deactivation steps (Rieke and Baylor, 1998). However, more recent work showed that a broad distribution of R^∗^ lifetimes (“noisy” rhodopsin) can be overcome by calcium feedback and generate reproducible SPRs. The very mechanism that explains “amplitude stability” of the SPR across genotypes also explains why late-stage R^∗^ deactivation steps do not decrease SPR reproducibility: specifically, when R^∗^ activity to be prolonged due to stochastic deactivation, delayed calcium feedback to cyclase ramps up to overcome the prolonged activity, stabilizing the WT SPR peak timing and amplitude. Modeling further reveals that, due to low-pass filtering of R^∗^ activity by the slow deactivation of E^∗^, trial-to-trial fluctuations in the brief lifetime of R^∗^ (∼40 ms) can account for the full degree of “late-stage” variability observed beyond the peak of the SPR (>130 ms; Gross et al., 2012a).

### MEMBRANE FLUX TRUMPS INTRACELLULAR DIFFUSION FOR INTRACELLULAR CALCIUM DYNAMICS

**FIGURE 3:**
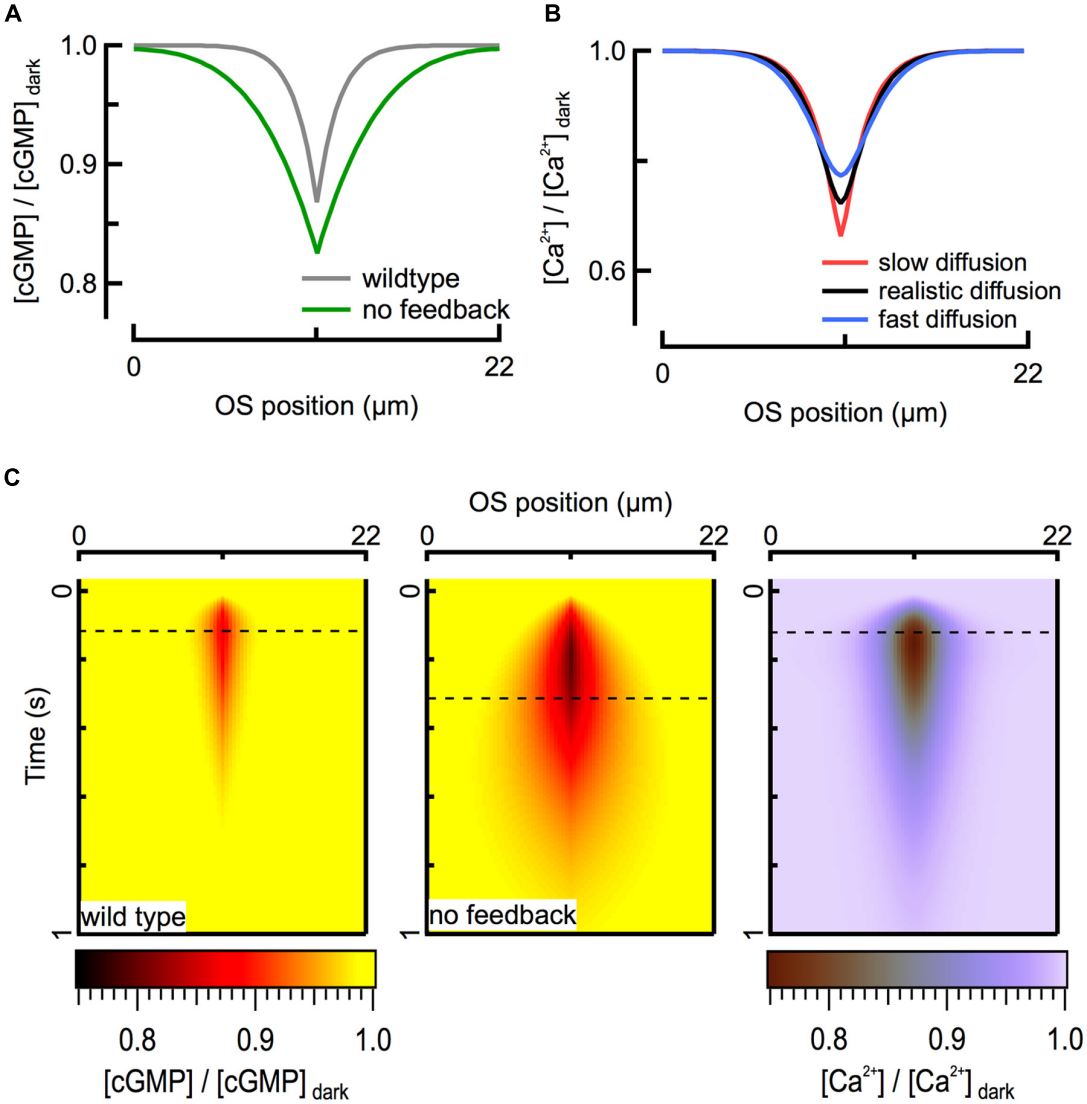
**Ca^**2+**^ feedback to cGMP synthesis restricts the spread of second messenger signals. (A)** Calculated spatial profiles of cGMP decline at the time of the peak of the WT (125 ms; gray) and GCAPs^-/-^ (320 ms; green) SPRs for an R^∗^ located in the middle of the rod (x = 11 μm). **(B)** The fall in intracellular Ca^2+^ is largely determined by the flux through CNG channels and is therefore insensitive to the value of the diffusion coefficient over the range 0.1 (red) – 10 (blue) μm^2^ s^-1^. Black profile represents *D*_Ca_ = 2 μm^2^ s^-1^. All traces correspond to the peak of the WT SPR. **(C)** Heat map representation of calculated cGMP spatiotemporal profiles during WT (*left*) and GCAPs^-/-^ (*center*) SPRs. The corresponding Ca^2+^ profile (*right*) is shown for the WT SPR. Dashed lines indicate time of peak response amplitudes.

For mouse rods with normal Ca^2+^ feedback to cyclase, theoretical calculations suggest that the spatial extent of the fall in calcium concentration largely mirrors that of cGMP (**Figure 3A**). Interestingly, this similarity in spatial profiles is largely independent of the value of the axial diffusion coefficient for calcium (*D*_Ca_) over the full range (0.1–10 μm^2^ s^-1^) of physiologically plausible values, given relatively strong intracellular calcium buffering (**Figure 3B**). While the maximal depth of the calcium decline is slightly altered when *D*_Ca_ is varied, the amplitude and time course of the simulated outer segment current response are not. The calcium profile is insensitive to *D*_Ca_ because the local net flux of Ca^2+^ through CNG channels and NCKX, which depends on the concentration gradient of calcium across the cell membrane, is much larger than what can be achieved through passive diffusion, given the relatively modest intracellular concentration gradient. To a first approximation, the magnitude of the exchange current is comparable to that of the Ca^2+^ component of the CNG channel current after a brief delay (Lagnado et al., 1992). Because the CNG channel current is determined exclusively by cGMP concentration, calcium concentration is also determined by the cGMP concentration (**Figure 3C**). Thus the diffusion barriers present in the outer segment directly influence only the spatio-temporal dynamics of cGMP, and the spatio-temporal dynamics of cGMP determine the distribution of calcium during a light response. Apparently, the calcium feedback system provides a form of self-restriction for the intracellular spread of the second messenger signal, contributing to linearity of the response by limiting overlap of signaling domains originating from multiple rhodopsin isomerizations.

## SUMMARY

Application of biochemical, electrophysiological, and computational approaches to understanding the spatio-temporal dynamics of cGMP signaling in vertebrate rods has yielded very good general agreement about the biochemical rates and local diffusional constraints underlying the amplitude, time course, and reproducibility of the SPR. The unification of our understanding across different genetic perturbations in mice (e.g., Gross et al., 2012a) and across vertebrate species (e.g., Reingruber et al., 2013) is encouraging, but there is still a great deal of work to be done. It is important to note that the single photon detection regime of rods constitutes only about three of the six orders of magnitude of intensities over which rods contribute to vision (e.g., Naarendorp et al., 2010). With higher light intensities come fundamental changes in biochemical parameters, cGMP and Ca^2+^ buffering, feedback mechanisms and cGMP dynamics. Future work will aim to build upon our understanding of spatiotemporal dynamics of cGMP signaling in dark-adapted rods by extending existing models to encompass known and new mechanisms of adaptation.

## Conflict of Interest Statement

The authors declare that the research was conducted in the absence of any commercial or financial relationships that could be construed as a potential conflict of interest.

## References

[B1] Ames J. B. Dizhoor A. M. Ikura M. Palczewski K. Stryer L. (1999). Three-dimensional structure of guanylyl cyclase activating protein-2, a calcium-sensitive modulator of photoreceptor guanylyl cyclases. *J. Biol. Chem.* 274 19329–19337 10.1074/jbc.274.27.19329 10383444

[B2] Andreucci D. Bisegna P. Caruso G. Hamm H. E. Dibenedetto E. (2003). Mathematical model of the spatio-temporal dynamics of second messengers in visual transduction. *Biophys. J.* 85 1358–1376 10.1016/S0006-3495(03)74570-6 12944255 PMC1303314

[B3] Baylor D. A. Lamb T. D. Yau K. W. (1979a). The membrane current of single rod outer segments. *J. Physiol.* 288 589–611. 112242 PMC1281446

[B4] Baylor D. A. Lamb T. D. Yau K. W. (1979b). Responses of retinal rods to single photons. *J. Physiol.* 288 613–634. 112243 PMC1281447

[B5] Baylor D. A. Matthews G. Yau K. W. (1980). Two components of electrical dark noise in toad retinal rod outer segments. *J. Physiol.* 309 591–621 10.1113/jphysiol.1980.sp013529 6788941 PMC1274605

[B6] Baylor D. A. Nunn B. J. (1986). Electrical properties of the light-sensitive conductance of rods of the salamander *Ambystoma tigrinum*. *J. Physiol.* 371 115–145 10.1113/jphysiol.1986.sp015964 2422346 PMC1192713

[B7] Baylor D. A. Nunn B. J. Schnapf J. L. (1984). The photocurrent, noise and spectral sensitivity of rods of the monkey *Macaca fascicularis*. *J. Physiol.* 357 575–607 10.1113/jphysiol.1984.sp015518 6512705 PMC1193276

[B8] Bisegna P. Caruso G. Andreucci D. Shen L. Gurevich V. V. Hamm H. E. (2008). Diffusion of the second messengers in the cytoplasm acts as a variability suppressor of the single photon response in vertebrate phototransduction. *Biophys. J.* 94 3363–3383 10.1529/biophysj.107.114058 18400950 PMC2292384

[B9] Bodoia R. D. Detwiler P. B. (1985). Patch-clamp recordings of the light-sensitive dark noise in retinal rods from the lizard and frog. *J. Physiol.* 367 183–216 10.1113/jphysiol.1985.sp015820 3877161 PMC1193059

[B10] Boyett M. R. Honjo H. Kodama I. (2000). The sinoatrial node, a heterogeneous pacemaker structure. *Cardiovasc. Res.* 47 658–687 10.1016/S0008-6363(00)00135-8 10974216

[B11] Burns M. E. Mendez A. Chen J. Baylor D. A. (2002). Dynamics of cyclic GMP synthesis in retinal rods. *Neuron* 36 81–91 10.1016/S0896-6273(02)00911-X 12367508

[B12] Burns M. E. Pugh E. N. Jr. (2010). Lessons from photoreceptors: turning off g-protein signaling in living cells. *Physiology (Bethesda)* 25 72–84 10.1152/physiol.00001.2010 20430952 PMC2880230

[B13] Burns M. E. Pugh E. N. Jr. (2014). “Visual transduction by rod and cone photoreceptors,” in *The New Visual Neurosciences* eds Werner J. S. Chalupa L. M. (Cambridge, MA: The MIT Press) 7–18.

[B14] Cameron D. A. Pugh E. N. Jr. (1990). The magnitude, time course and spatial distribution of current induced in salamander rods by cyclic guanine nucleotides. *J. Physiol.* 430 419–439 10.1113/jphysiol.1990.sp018299 1964967 PMC1181745

[B15] Caruso G. Bisegna P. Andreucci D. Lenoci L. Gurevich V. V. Hamm H. E. (2011). Identification of key factors that reduce the variability of the single photon response. *Proc. Natl. Acad. Sci. U.S.A.* 108 7804–7807 10.1073/pnas.1018960108 21518901 PMC3093507

[B16] Caruso G. Bisegna P. Lenoci L. Andreucci D. Gurevich V. V. Hamm H. E. (2010). Kinetics of rhodopsin deactivation and its role in regulating recovery and reproducibility of rod photoresponse. *PLoS Comput. Biol.* 6:e1001031 10.1371/journal.pcbi.1001031 PMC3002991 21200415

[B17] Cobbs W. H. Pugh E. N. Jr. (1987). Kinetics and components of the flash photocurrent of isolated retinal rods of the larval salamander, *Ambystoma tigrinum*. *J. Physiol.* 394 529–572 10.1113/jphysiol.1987.sp016884 2832596 PMC1191975

[B18] Cohen A. I. (1963). Vertebrate retinal cells and their organization. *Biol. Rev.* 38 427–459 10.1111/j.1469-185X.1963.tb00789.x

[B19] Craven K. B. Zagotta W. N. (2006). CNG and HCN channels: two peas, one pod. *Annu. Rev. Physiol.* 68 375–401 10.1146/annurev.physiol.68.040104.134728 16460277

[B20] Dizhoor A. M. Hurley J. B. (1996). Inactivation of EF-hands makes GCAP-2 (p24) a constitutive activator of photoreceptor guanylyl cyclase by preventing a Ca^2+^-induced “activator-to-inhibitor” transition. *J. Biol. Chem.* 271 19346–19350 10.1074/jbc.271.32.19346 8702620

[B21] Dizhoor A. M. Hurley J. B. (1999). Regulation of photoreceptor membrane guanylyl cyclases by guanylyl cyclase activator proteins. *Methods* 19 521–531 10.1006/meth.1999.0894 10581151

[B22] Dizhoor A. M. Olshevskaya E. V. Peshenko I. V. (2010). Mg^2+^/Ca^2+^ cation binding cycle of guanylyl cyclase activating proteins (GCAPs): role in regulation of photoreceptor guanylyl cyclase. *Mol. Cell. Biochem.* 334 117–124 10.1007/s11010-009-0328-6 19953307 PMC2824334

[B23] Fesenko E. E. Kolesnikov S. S. Lyubarsky A. L. (1985). Induction by cyclic GMP of cationic conductance in plasma membrane of retinal rod outer segment. *Nature* 313 310–313 10.1038/313310a0 2578616

[B24] Field G. D. Rieke F. (2002). Mechanisms regulating variability of the single photon responses of mammalian rod photoreceptors. *Neuron* 35 733–747 10.1016/S0896-6273(02)00822-X 12194872

[B25] Fu Y. Yau K. W. (2007). Phototransduction in mouse rods and cones. *Pflugers. Arch.* 454 805–819 10.1007/s00424-006-0194-y 17226052 PMC2877390

[B26] Gray-Keller M. Denk W. Shraiman B. Detwiler P. B. (1999). Longitudinal spread of second messenger signals in isolated rod outer segments of lizards. *J. Physiol.* 519(Pt 3), 679–692 10.1111/j.1469-7793.1999.0679n.x 10457083 PMC2269547

[B27] Gross O. P. Burns M. E. (2010). Control of rhodopsin’s active lifetime by arrestin-1 expression in mammalian rods. *J. Neurosci.* 30 3450–3457 10.1523/JNEUROSCI.5391-09.2010 20203204 PMC2841010

[B28] Gross O. P. Pugh E. N. Jr. Burns M. E. (2012a). Calcium feedback to cGMP synthesis strongly attenuates single-photon responses driven by long rhodopsin lifetimes. *Neuron* 76 370–382 10.1016/j.neuron.2012.07.029 23083739 PMC3594095

[B29] Gross O. P. Pugh E. N. Jr. Burns M. E. (2012b). Spatiotemporal cGMP dynamics in living mouse rods. *Biophys. J.* 102 1775–1784 10.1016/j.bpj.2012.03.035 22768933 PMC3328695

[B30] Hamer R. D. Nicholas S. C. Tranchina D. Liebman P. A. Lamb T. D. (2003). Multiple steps of phosphorylation of activated rhodopsin can account for the reproducibility of vertebrate rod single-photon responses. *J. Gen. Physiol.* 122 419–444 10.1085/jgp.200308832 12975449 PMC1480412

[B31] Haynes L. W. Kay A. R. Yau K. W. (1986). Single cyclic GMP-activated channel activity in excised patches of rod outer segment membrane. *Nature* 321 66–70 10.1038/321066a0 2422558

[B32] Heck M. Hofmann K. P. (2001). Maximal rate and nucleotide dependence of rhodopsin-catalyzed transducin activation: initial rate analysis based on a double displacement mechanism. *J. Biol. Chem.* 276 10000–10009 10.1074/jbc.M009475200 11116153

[B33] Hemilä S. Reuter T. (1981). Longitudinal spread of adaptation in the rods of the frog’s retina. *J. Physiol.* 310 501–528 10.1113/jphysiol.1981.sp013564 6971931 PMC1274755

[B34] Hodgkin A. L. Mcnaughton P. A. Nunn B. J. (1987). Measurement of sodium-calcium exchange in salamander rods. *J. Physiol.* 391 347–370 10.1113/jphysiol.1987.sp016742 2451008 PMC1192218

[B35] Holcman D. Korenbrot J. I. (2004). Longitudinal diffusion in retinal rod and cone outer segment cytoplasm: the consequence of cell structure. *Biophys. J.* 86 2566–2582 10.1016/S0006-3495(04)74312-X 15041693 PMC1304104

[B36] Karpen J. W. Zimmerman A. L. Stryer L. Baylor D. A. (1988). Gating kinetics of the cyclic-GMP-activated channel of retinal rods: flash photolysis and voltage-jump studies. *Proc. Natl. Acad. Sci. U.S.A.* 85 1287–1291 10.1073/pnas.85.4.1287 2448798 PMC279752

[B37] Koch K. W. Stryer L. (1988). Highly cooperative feedback control of retinal rod guanylate cyclase by calcium ions. *Nature* 334 64–66 10.1038/334064a0 2455233

[B38] Korenbrot J. I. Miller D. L. (1989). Cytoplasmic free calcium concentration in dark-adapted retinal rod outer segments. *Vision Res.* 29 939–948 10.1016/0042-6989(89)90108-9 2516928

[B39] Korschen H. G. Beyermann M. Muller F. Heck M. Vantler M. Koch K. W. (1999). Interaction of glutamic-acid-rich proteins with the cGMP signalling pathway in rod photoreceptors. *Nature* 400 761–766 10.1038/23468 10466724

[B40] Koutalos Y. Brown R. L. Karpen J. W. Yau K.-W. (1995a). Diffusion coefficient of the cyclic GMP analog 8-(fluoresceinyl)thioguanosine 3′,5′ cyclic monophosphate in the salamander rod outer segment. *Biophys. J.* 69 2163–2167 10.1016/S0006-3495(95)80090-1 8580360 PMC1236450

[B41] Koutalos Y. Nakatani K. Tamura T. Yau K. W. (1995b). Characterization of guanylate cyclase activity in single retinal rod outer segments. *J. Gen. Physiol.* 106 863–890 10.1085/jgp.106.5.863 8648296 PMC2229293

[B42] Koutalos Y. Nakatani K. Yau K.-W. (1995c). Cyclic GMP diffusion coefficient in rod photoreceptor outer segments. *Biophys. J.* 68 373–382 10.1016/S0006-3495(95)80198-0 7536055 PMC1281697

[B43] Krispel C. M. Chen D. Melling N. Chen Y. J. Martemyanov K. A. Quillinan N. (2006). RGS expression rate-limits recovery of rod photoresponses. *Neuron* 51 409–416 10.1016/j.neuron.2006.07.010 16908407

[B44] Lagnado L. Cervetto L. Mcnaughton P. A. (1992). Calcium homeostasis in the outer segments of retinal rods from the tiger salamander. *J. Physiol.* 455 111–142 10.1113/jphysiol.1992.sp019293 1282928 PMC1175636

[B45] Lamb T. D. Mcnaughton P. A. Yau K. W. (1981). Spatial spread of activation and background desensitization in toad rod outer segments. *J. Physiol.* 319 463–496 10.1113/jphysiol.1981.sp013921 6798202 PMC1243851

[B46] Lamb T. D. Pugh E. N. Jr. (1992). A quantitative account of the activation steps involved in phototransduction in amphibian photoreceptors. *J. Physiol.* 449 719–758 10.1113/jphysiol.1992.sp019111 1326052 PMC1176104

[B47] Leskov I. B. Klenchin V. A. Handy J. W. Whitlock G. G. Govardovskii V. I. Bownds M. D. (2000). The gain of rod phototransduction: reconciliation of biochemical and electrophysiological measurements. *Neuron* 27 525–537 10.1016/S0896-6273(00)00063-5 11055435

[B48] Makino C. L. Dodd R. L. Chen J. Burns M. E. Roca A. Simon M. I. (2004). Recoverin regulates light-dependent phosphodiesterase activity in retinal rods. *J. Gen. Physiol.* 123 729–741 10.1085/jgp.200308994 15173221 PMC2234569

[B49] Makino C. L. Peshenko I. V. Wen X. H. Olshevskaya E. V. Barrett R. Dizhoor A. M. (2008). A role for GCAP2 in regulating the photoresponse. Guanylyl cyclase activation and rod electrophysiology in GUCA1B knock-out mice. *J. Biol. Chem.* 283 29135–29143 10.1074/jbc.M804445200 18723510 PMC2570858

[B50] Mendez A. Burns M. E. Sokal I. Dizhoor A. M. Baehr W. Palczewski K. (2001). Role of guanylate cyclase-activating proteins (GCAPs) in setting the flash sensitivity of rod photoreceptors. *Proc. Natl. Acad. Sci. U.S.A.* 98 9948–9953 10.1073/pnas.171308998 11493703 PMC55558

[B51] Naarendorp F. Esdaille T. M. Banden S. M. Andrews-Labenski J. Gross O. P. Pugh E. N. Jr. (2010). Dark light, rod saturation, and the absolute and incremental sensitivity of mouse cone vision. *J. Neurosci.* 30 12495–12507 10.1523/JNEUROSCI.2186-10.2010 20844144 PMC3423338

[B52] Nikonov S. Lamb T. D. Pugh E. N. Jr. (2000). The role of steady phosphodiesterase activity in the kinetics and sensitivity of the light-adapted salamander rod photoresponse. *J. Gen. Physiol.* 116 795–824 10.1085/jgp.116.6.795 11099349 PMC2231811

[B53] Olson A. Pugh E. N. Jr. (1993). Diffusion coefficient of cyclic GMP in salamander rod outer segments estimated with two fluorescent probes. *Biophys. J.* 65 1335–1352 10.1016/S0006-3495(93)81177-9 8241412 PMC1225852

[B54] Palczewski K. Polans A. S. Baehr W. Ames J. B. (2000). Ca(2+)-binding proteins in the retina: structure, function, and the etiology of human visual diseases. *Bioessays* 22 337–350 10.1002/(SICI)1521-1878(200004)22:4<337::AID-BIES4>3.0.CO;2-Z 10723031

[B55] Palczewski K. Subbaraya I. Gorczyca W. A. Helekar B. S. Ruiz C. C. Ohguro H. (1994). Molecular cloning and characterization of retinal photoreceptor guanylyl cyclase-activating protein. *Neuron* 13 395–404 10.1016/0896-6273(94)90355-7 7520254

[B56] Peshenko I. V. Dizhoor A. M. (2004). Guanylyl cyclase-activating proteins (GCAPs) are Ca^2+^/Mg^2+^ sensors: implications for photoreceptor guanylyl cyclase (RetGC) regulation in mammalian photoreceptors. *J. Biol. Chem.* 279 16903–16906 10.1074/jbc.C400065200 14993224

[B57] Peshenko I. V. Olshevskaya E. V. Savchenko A. B. Karan S. Palczewski K. Baehr W. (2011). Enzymatic properties and regulation of the native isozymes of retinal membrane guanylyl cyclase (RetGC) from mouse photoreceptors. *Biochemistry* 50 5590–5600 10.1021/bi200491b 21598940 PMC3127287

[B58] Poetsch A. Molday L. L. Molday R. S. (2001). The cGMP-gated channel and related glutamic acid-rich proteins interact with peripherin-2 at the rim region of rod photoreceptor disc membranes. *J. Biol. Chem.* 276 48009–48016. 11641407 10.1074/jbc.M108941200

[B59] Pugh E. N. Jr. Lamb T. D. (1993). Amplification and kinetics of the activation steps in phototransduction. *Biochim. Biophys. Acta* 1141 111–149 10.1016/0005-2728(93)90038-H 8382952

[B60] Reingruber J. Holcman D. (2008). Estimating the rate constant of cyclic GMP hydrolysis by activated phosphodiesterase in photoreceptors. *J. Chem. Phys.* 129:145102 10.1063/1.2991174 19045167

[B61] Reingruber J. R. Pahlberg J. Woodruff M. L. Sampath A. P. Fain G. L. Holcman D. (2013). Detection of single photons by toad and mouse rods. *Proc. Natl. Acad. Sci. U.S.A.* 110 19378–19383 10.1073/pnas.1314030110 24214653 PMC3845110

[B62] Rieke F. Baylor D. A. (1996). Molecular origin of continuous dark noise in rod photoreceptors. *Biophys. J.* 71 2553–2572 10.1016/S0006-3495(96)79448-1 8913594 PMC1233743

[B63] Rieke F. Baylor D. A. (1998). Origin of reproducibility in the responses of retinal rods to single photons. *Biophys. J.* 75 1836–1857 10.1016/S0006-3495(98)77625-8 9746525 PMC1299855

[B64] Ritter L. M. Khattree N. Tam B. Moritz O. L. Schmitz F. Goldberg A. F. (2011). In situ visualization of protein interactions in sensory neurons: glutamic acid-rich proteins (GARPs) play differential roles for photoreceptor outer segment scaffolding. *J. Neurosci.* 31 11231–11243 10.1523/JNEUROSCI.2875-11.2011 21813684 PMC3158677

[B65] Ruiz M. Brown R. L. He Y. Haley T. L. Karpen J. W. (1999). The single-channel dose-response relation is consistently steep for rod cyclic nucleotide-gated channels: implications for the interpretation of macroscopic dose-response relations. *Biochemistry* 38 10642–10648 10.1021/bi990532w 10451358

[B66] Sampath A. P. Matthews H. R. Cornwall M. C. Fain G. L. (1998). Bleached pigment produces a maintained decrease in outer segment Ca^2+^ in salamander rods. *J. Gen. Physiol.* 111 53–64 10.1085/jgp.111.1.53 9417134 PMC1887770

[B67] Schwarzer A. Schauf H. Bauer P. J. (2000). Binding of the cGMP-gated channel to the Na/Ca-K exchanger in rod photoreceptors. *J. Biol. Chem.* 275 13448–13454 10.1074/jbc.275.18.13448 10788457

[B68] Sharma R. K. (2010). Membrane guanylate cyclase is a beautiful signal transduction machine: overview. *Mol. Cell. Biochem.* 334 3–36 10.1007/s11010-009-0336-6 19957201

[B69] Shuart N. G. Haitin Y. Camp S. S. Black K. D. Zagotta W. N. (2011). Molecular mechanism for 3:1 subunit stoichiometry of rod cyclic nucleotide-gated ion channels. *Nat. Commun.* 2 457–457 10.1038/ncomms1466 21878911 PMC3265371

[B70] Tao T. Paterson D. J. Smith N. P. (2011). A model of cellular cardiac-neural coupling that captures the sympathetic control of sinoatrial node excitability in normotensive and hypertensive rats. *Biophys. J.* 101 594–602 10.1016/j.bpj.2011.05.069 21806927 PMC3145287

[B71] Whitlock G. G. Lamb T. D. (1999). Variability in the time course of single photon responses from toad rods: termination of rhodopsin’s activity. *Neuron* 23 337–351 10.1016/S0896-6273(00)80784-9 10399939

[B72] Woodruff M. L. Sampath A. P. Matthews H. R. Krasnoperova N. V. Lem J. Fain G. L. (2002). Measurement of cytoplasmic calcium concentration in the rods of wild-type and transducin knock-out mice. *J. Physiol.* 542 843–854 10.1113/jphysiol.2001.013987 12154183 PMC2290451

[B73] Wu Q. Chen C. Koutalos Y. (2006). Longitudinal diffusion of a polar tracer in the outer segments of rod photoreceptors from different species. *Photochem. Photobiol.* 82 1447–1451 10.1562/2006-02-22-RA-807 16906792

